# Association of State-Issued Mask Mandates and Allowing On-Premises Restaurant Dining with County-Level COVID-19 Case and Death Growth Rates — United States, March 1–December 31, 2020

**DOI:** 10.15585/mmwr.mm7010e3

**Published:** 2021-03-12

**Authors:** Gery P. Guy, Florence C. Lee, Gregory Sunshine, Russell McCord, Mara Howard-Williams, Lyudmyla Kompaniyets, Christopher Dunphy, Maxim Gakh, Regen Weber, Erin Sauber-Schatz, John D. Omura, Greta M. Massetti, CDC COVID-19 Response Team, Mitigation Policy Analysis Unit, CDC Public Health Law Program, CDC COVID-19 Response Team, Mitigation Policy Analysis Unit, Moriah Bailey, Amanda Brown, Ryan Cramer, Catherine Clodfelter, Robin Davison, Sebnem Dugmeoglu, Arriana Fitts, Siobhan Gilchrist, Rachel Hulkower, Alexa Limeres, Dawn Pepin, Adebola Popoola, Morgan Schroeder, Michael A. Tynan, Chelsea Ukoha, Michael Williams, Christopher D. Whitson, CDC Public Health Law Program, Gi Jeong, Lisa Landsman, Amanda Moreland, Julia Shelburne

**Affiliations:** ^1^CDC COVID-19 Response Team; ^2^CDC Public Health Law Program; ^3^University of Nevada, Las Vegas.; CDC; CDC; CDC; CDC; CDC; CDC; CDC; CDC; CDC; CDC; CDC; CDC; CDC; CDC; CDC; CDC; CDC.; CDC; CDC; CDC; CDC.

CDC recommends a combination of evidence-based strategies to reduce transmission of SARS-CoV-2, the virus that causes COVID-19 (*1*). Because the virus is transmitted predominantly by inhaling respiratory droplets from infected persons, universal mask use can help reduce transmission (*1*). Starting in April, 39 states and the District of Columbia (DC) issued mask mandates in 2020. Reducing person-to-person interactions by avoiding nonessential shared spaces, such as restaurants, where interactions are typically unmasked and physical distancing (≥6 ft) is difficult to maintain, can also decrease transmission (*2*). In March and April 2020, 49 states and DC prohibited any on-premises dining at restaurants, but by mid-June, all states and DC had lifted these restrictions. To examine the association of state-issued mask mandates and allowing on-premises restaurant dining with COVID-19 cases and deaths during March 1–December 31, 2020, county-level data on mask mandates and restaurant reopenings were compared with county-level changes in COVID-19 case and death growth rates relative to the mandate implementation and reopening dates. Mask mandates were associated with decreases in daily COVID-19 case and death growth rates 1–20, 21–40, 41–60, 61–80, and 81–100 days after implementation. Allowing any on-premises dining at restaurants was associated with increases in daily COVID-19 case growth rates 41–60, 61–80, and 81–100 days after reopening, and increases in daily COVID-19 death growth rates 61–80 and 81–100 days after reopening. Implementing mask mandates was associated with reduced SARS-CoV-2 transmission, whereas reopening restaurants for on-premises dining was associated with increased transmission. Policies that require universal mask use and restrict any on-premises restaurant dining are important components of a comprehensive strategy to reduce exposure to and transmission of SARS-CoV-2 (*1*). Such efforts are increasingly important given the emergence of highly transmissible SARS-CoV-2 variants in the United States (*3*,*4*).

County-level data on state-issued mask mandates and restaurant closures were obtained from executive and administrative orders identified on state government websites. Orders were analyzed and coded to extract mitigation policy variables for mask mandates and restaurant closures, their effective dates and expiration dates, and the counties to which they applied. State-issued mask mandates were defined as requirements for persons to wear a mask 1) anywhere outside their home or 2) in retail businesses and in restaurants or food establishments. State-issued restaurant closures were defined as prohibitions on restaurants operating or limiting service to takeout, curbside pickup, or delivery. Allowing restaurants to provide indoor or outdoor on-premises dining was defined as the state lifting a state-issued restaurant closure.* All coding underwent secondary review and quality assurance checks by two or more raters; upon agreement among all raters, coding and analyses were published in freely available data sets.^†^^,^^§^

Two outcomes were examined: the daily percentage point growth rate of county-level COVID-19 cases and county-level COVID-19 deaths. The daily growth rate was defined as the difference between the natural log of cumulative cases or deaths on a given day and the natural log of cumulative cases or deaths on the previous day, multiplied by 100. Data on cumulative county-level COVID-19 cases and deaths were collected from state and local health department websites and accessed through U.S. Department of Health and Human Services Protect.^¶^

Associations between the policies and COVID-19 outcomes were measured using a reference period (1–20 days before implementation) compared with seven mutually exclusive time ranges relative to implementation (i.e., the effective date of the mask mandate or the date restaurants were permitted to allow on-premises dining). The association was examined over two preimplementation periods (60–41 and 40–21 days before implementation) and five postimplementation periods (1–20, 21–40, 41–60, 61–80, and 81–100 days after implementation).

Weighted least-squares regression with county and day fixed effects was used to compare COVID-19 case and death growth rates before and after 1) implementing mask mandates and 2) allowing on-premises dining at restaurants. Because state-issued policies often applied to specific counties, particularly when states began allowing on-premises dining, all analyses were conducted at the county level. Four regression models were used to assess the association between each policy and each COVID-19 outcome. The regression models controlled for several covariates: restaurant closures in the mask mandate models and mask mandates in the restaurant reopening models, as well as bar closures,** stay-at-home orders,^††^ bans on gatherings of ≥10 persons,^§§^ daily COVID-19 tests per 100,000 persons, county, and time (day). P-values <0.05 were considered statistically significant. All analyses were weighted by county population with standard errors robust to heteroscedasticity and clustered by state. Analyses were performed using Stata software (version 14.2; StataCorp). This activity was reviewed by CDC and was conducted consistent with applicable federal law and CDC policy.^¶¶^

During March 1–December 31, 2020, state-issued mask mandates applied in 2,313 (73.6%) of the 3,142 U.S. counties. Mask mandates were associated with a 0.5 percentage point decrease (p = 0.02) in daily COVID-19 case growth rates 1–20 days after implementation and decreases of 1.1, 1.5, 1.7, and 1.8 percentage points 21–40, 41–60, 61–80, and 81–100 days, respectively, after implementation (p<0.01 for all) (Table 1) (Figure). Mask mandates were associated with a 0.7 percentage point decrease (p = 0.03) in daily COVID-19 death growth rates 1–20 days after implementation and decreases of 1.0, 1.4, 1.6, and 1.9 percentage points 21–40, 41–60, 61–80, and 81–100 days, respectively, after implementation (p<0.01 for all). Daily case and death growth rates before implementation of mask mandates were not statistically different from the reference period.

**TABLE 1:** Association between state-issued mask mandates* and changes in COVID-19 case and death growth rates^†^ — United States, March 1–December 31, 2020

Time relative to day state mask mandate was implemented	Case growth rates		Death growth rates	
	Percentage point change (95% CI)	p-value^§^	Percentage point change (95% CI)	p-value^§^
41–60 days before	0.0 (−0.7 to 0.7)	0.98	−0.8 (−1.8 to 0.1)	0.07
21–40 days before	0.5 (−0.8 to 1.8)	0.49	0.3 (−0.8 to 1.5)	0.56
1–20 days before	Referent	—	Referent	—
1–20 days after	−0.5 (−0.8 to −0.1)	0.02	−0.7 (−1.4 to −0.1)	0.03
21–40 days after	−1.1 (−1.6 to −0.6)	<0.01	−1.0 (−1.7 to −0.3)	<0.01
41–60 days after	−1.5 (−2.1 to −0.8)	<0.01	−1.4 (−2.2 to −0.6)	<0.01
61–80 days after	−1.7 (−2.6 to −0.9)	<0.01	−1.6 (−2.4 to −0.7)	<0.01
81–100 days after	−1.8 (−2.8 to −0.7)	<0.01	−1.9 (−3.0 to −0.8)	<0.01

**Abbreviation:** CI = confidence interval.

* A state-issued mask mandate was defined as the requirement that persons operating in a personal capacity (i.e., not limited to specific professions or employees) wear a mask 1) anywhere outside their home or 2) in retail businesses and in restaurants or food establishments.

^†^ Percentage points are coefficients from the weighted least-squares regression models. Reported numbers are from regression models, which controlled for county, time (day), COVID-19 tests per 100,000 persons, closure of restaurants for any on-premises dining, closure of bars for any on-premises dining, and the presence of gathering bans and stay-at-home orders.

**^§^** P-values <0.05 were considered statistically significant.

**FIGURE:**
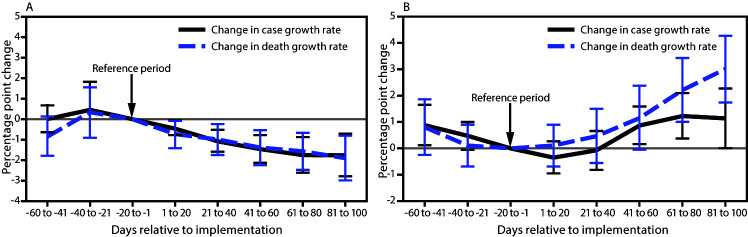
Association between changes in COVID-19 case and death growth rates* and implementation of state mask mandates^†^ (A) and states allowing any on-premises restaurant dining^§^ (B) — United States, March 1–December 31, 2020 * With 95% confidence intervals indicated with error bars. ^†^ A state-issued mask mandate was defined as the requirement that persons operating in a personal capacity (i.e., not limited to specific professions or employees) wear a mask 1) anywhere outside their home or 2) in retail businesses and in restaurants or food establishments. **^§^** The effective date of the state order allowing restaurants to conduct any on-premises dining or the date a state-issued restaurant closure expired.

During the study period, states allowed restaurants to reopen for on-premises dining in 3,076 (97.9%) U.S. counties. Changes in daily COVID-19 case and death growth rates were not statistically significant 1–20 and 21–40 days after restrictions were lifted. Allowing on-premises dining at restaurants was associated with 0.9 (p = 0.02), 1.2 (p<0.01), and 1.1 (p = 0.04) percentage point increases in the case growth rate 41–60, 61–80, and 81–100 days, respectively, after restrictions were lifted (Table 2) (Figure). Allowing on-premises dining at restaurants was associated with 2.2 and 3.0 percentage point increases in the death growth rate 61–80 and 81–100 days, respectively, after restrictions were lifted (p<0.01 for both). Daily death growth rates before restrictions were lifted were not statistically different from those during the reference period, whereas significant differences in daily case growth rates were observed 41–60 days before restrictions were lifted.

**TABLE 2:** Association between states allowing any on-premises restaurant dining* and changes in COVID-19 case and death growth rates^†^ — United States, March 1–December 31, 2020

Time relative to day states allowed on-premises dining	Case growth rates		Death growth rates	
	Percentage point change (95% CI)	p-value^§^	Percentage point change (95% CI)	p-value^§^
41–60 days before	0.9 (0.1 to 1.6)	0.02	0.8 (−0.2 to 1.8)	0.13
21–40 days before	0.5 (−0.1 to 1.0)	0.08	0.1 (−0.7 to 0.9)	0.78
1–20 days before	Referent	—	Referent	—
1–20 days after	−0.4 (−0.9 to 0.2)	0.22	0.1 (−0.7 to 0.9)	0.78
21–40 days after	−0.1 (−0.8 to 0.6)	0.83	0.5 (−0.5 to 1.5)	0.36
41–60 days after	0.9 (0.2 to 1.6)	0.02	1.1 (−0.1 to 2.3)	0.06
61–80 days after	1.2 (0.4 to 2.1)	<0.01	2.2 (1.0 to 3.4)	<0.01
81–100 days after	1.1 (0.0 to 2.2)	0.04	3.0 (1.8 to 4.3)	<0.01

**Abbreviation:** CI = confidence interval.

* The effective date of the state order allowing restaurants to conduct any on-premises dining or the date a state-issued restaurant closure expired.

^†^ Percentage points are coefficients from the weighted least-squares regression models. Reported numbers are from regression models, which controlled for county, time (day), COVID-19 tests per 100,000 persons, mask mandates, closure of bars for any on-premises dining, and the presence of gathering bans and stay-at-home orders.

**^§^** P-values <0.05 were considered statistically significant.

## Discussion

Mask mandates were associated with statistically significant decreases in county-level daily COVID-19 case and death growth rates within 20 days of implementation. Allowing on-premises restaurant dining was associated with increases in county-level case and death growth rates within 41–80 days after reopening. State mask mandates and prohibiting on-premises dining at restaurants help limit potential exposure to SARS-CoV-2, reducing community transmission of COVID-19.

Studies have confirmed the effectiveness of community mitigation measures in reducing the prevalence of COVID-19 (*5*–*8*). Mask mandates are associated with reductions in COVID-19 case and hospitalization growth rates (*6*,*7*), whereas reopening on-premises dining at restaurants, a known risk factor associated with SARS-CoV-2 infection (*2*), is associated with increased COVID-19 cases and deaths, particularly in the absence of mask mandates (*8*). The current study builds upon this evidence by accounting for county-level variation in state-issued mitigation measures and highlights the importance of a comprehensive strategy to decrease exposure to and transmission of SARS-CoV-2. Prohibiting on-premises restaurant dining might assist in limiting potential exposure to SARS-CoV-2; however, such orders might disrupt daily life and have an adverse impact on the economy and the food services industry (*9*). If on-premises restaurant dining options are not prohibited, CDC offers considerations for operators and customers which can reduce the risk of spreading COVID-19 in restaurant settings.*** COVID-19 case and death growth rates might also have increased because of persons engaging in close contact activities other than or in addition to on-premises restaurant dining in response to perceived reduced risk as a result of states allowing restaurants to reopen. Further studies are needed to assess the effect of a multicomponent community mitigation strategy on economic activity.

Increases in COVID-19 case and death growth rates were significantly associated with on-premises dining at restaurants after indoor or outdoor on-premises dining was allowed by the state for >40 days. Several factors might explain this observation. Even though prohibition of on-premises restaurant dining was lifted, restaurants were not required to open and might have delayed reopening. In addition, potential restaurant patrons might have been more cautious when restaurants initially reopened for on-premises dining but might have been more likely to dine at restaurants as time passed. Further analyses are necessary to evaluate the delayed increase in case and death growth rates.

The findings in this report are subject to at least three limitations. First, although models controlled for mask mandates, restaurant and bar closures, stay-at-home orders, and gathering bans, the models did not control for other policies that might affect case and death rates, including other types of business closures, physical distancing recommendations, policies issued by localities, and variances granted by states to certain counties if variances were not made publicly available. Second, compliance with and enforcement of policies were not measured. Finally, the analysis did not differentiate between indoor and outdoor dining, adequacy of ventilation, and adherence to physical distancing and occupancy requirements.

Community mitigation measures can help reduce the transmission of SARS-CoV-2. In this study, mask mandates were associated with reductions in COVID-19 case and death growth rates within 20 days, whereas allowing on-premises dining at restaurants was associated with increases in COVID-19 case and death growth rates after 40 days. With the emergence of more transmissible COVID-19 variants, community mitigation measures are increasingly important as part of a larger strategy to decrease exposure to and reduce transmission of SARS-CoV-2 (*3*,*4*). Community mitigation policies, such as state-issued mask mandates and prohibition of on-premises restaurant dining, have the potential to slow the spread of COVID-19, especially if implemented with other public health strategies (*1*,*10*).

**Summary**

**What is already known about this topic?**

Universal masking and avoiding nonessential indoor spaces are recommended to mitigate the spread of COVID-19.

**What is added by this report?**

Mandating masks was associated with a decrease in daily COVID-19 case and death growth rates within 20 days of implementation. Allowing on-premises restaurant dining was associated with an increase in daily COVID-19 case growth rates 41–100 days after implementation and an increase in daily death growth rates 61–100 days after implementation.

**What are the implications for public health practice?**

Mask mandates and restricting any on-premises dining at restaurants can help limit community transmission of COVID-19 and reduce case and death growth rates. These findings can inform public policies to reduce community spread of COVID-19.

## Footnotes

* For the purposes of this analysis, no distinction was made based on whether reopened restaurants were subject to state requirements to implement safety measures, such as limit dining to outdoor service, reduce capacity, enhance sanitation, or physically distance, or if no mandatory restrictions applied. When states differentiated between bars that serve food and bars that do not serve food, restrictions for bars that serve food were coded as restaurants and restrictions for bars that do not serve food were coded as bars.

† https://ephtracking.cdc.gov/DataExplorer/?c=33&i=165 (accessed February 24, 2021)

§ https://ephtracking.cdc.gov/DataExplorer/?c=33&i=162 (accessed February 24, 2021)

¶ https://protect-public.hhs.gov (accessed February 3, 2021)

** https://data.cdc.gov/Policy-Surveillance/U-S-State-and-Territorial-Orders-Closing-and-Reope/9kjw-3miq (accessed February 24, 2021)

†† https://data.cdc.gov/Policy-Surveillance/U-S-State-and-Territorial-Stay-At-Home-Orders-Marc/y2iy-8irm (accessed February 24, 2021)

§§ https://data.cdc.gov/Policy-Surveillance/U-S-State-and-Territorial-Gathering-Bans-March-11-/7xvh-y5vh (accessed February 24, 2021)

¶¶ 45 C.F.R. part 46, 21 C.F.R. part 56; 42 U.S.C. Sect. 241(d); 5 U.S.C. Sect. 552a; 44 U.S.C. Sect. 3501 et seq.

***
https://www.cdc.gov/coronavirus/2019-ncov/community/organizations/business-employers/bars-restaurants.html
