# *Erratum:* Vol. 70, No. 10

**DOI:** 10.15585/mmwr.mm7021a4

**Published:** 2021-05-28

In the report “Association of State-Issued Mask Mandates and Allowing On-Premises Restaurant Dining with County-Level COVID-19 Case and Death Growth Rates — United States, March 1–December 31, 2020,” on page 350, the third sentence in the first paragraph should have read, “Starting in April, **38** states and the District of Columbia (DC) issued mask mandates in 2020.”

